# The Effects of Behavioral Foundations and Business Strategy on Corporate Dividend Policy

**DOI:** 10.3389/fpsyg.2022.849238

**Published:** 2022-03-23

**Authors:** Wen-Ju Liao, Yu-En Lin, Xin-Zhe Li, Hsiang-Hsuan Chih

**Affiliations:** ^1^Newhuadu Business School, Minjiang University, Fuzhou, China; ^2^Center for Quantitative Economics, Jilin University, Changchun, China; ^3^School of Business, Renmin University of China, Beijing, China; ^4^Finance Department, National Dong Hwa University, Hualien, Taiwan

**Keywords:** dividend policy, ambiguity aversion, loss aversion, risk aversion, business strategy

## Abstract

This study analyzes the influence of behavioral foundation factors and corporate strategic behavior on the formulation of corporate dividend policy. We use the Logit model and the OLS model for estimating the empirical model. The year- and industry-fixed effects are controlled in the model. We consider the behavioral foundations in three dimensions-ambiguity aversions, risk aversion, and loss aversion. The results show firms with high ambiguity or high risk infrequently pay dividends but firms with loss-averse behavior tend to pay dividends. This paper also provides evidence that a firms’ business strategy influences its corporate dividend policy. Aggressive firms inhibit the payout of dividends. In additional tests, we find the results remain unchanged in those firms with high corporate governance or high growth opportunities.

## Introduction

Dividend policy, as one of the most critical corporate decisions, refers to the payout policy that a firm follows in determining whether to distribute dividends to shareholders and the size of the cash distributions over time. Numerous studies have focused on the determinants of dividends, but there is still no explanation widely accepted (Baker et al., 2011). Therefore, more efforts should be put in place to clarify the picture and uncover the “dividend puzzle” (Black, 1976).

One issue that contributes to the fuzzy picture of dividend policy and hinders the elaboration of new perspectives is the assumption that people are always rational and will pursue the maximization of personal profits. The rising studies consider behavioral biases in modeling complex decision-making processes (Agliardi et al., 2015). Baker and Wurgler (2004) point out investors’ trading behavior positively respond to dividend payouts, so managers tend to cater to investors for obtaining stock premiums (also see Li and Lie, 2006; Ferris et al., 2009). Several studies found that overconfident managers tend not to pay dividends or pay fewer cash dividends (Heaton, 2002; Malmendier et al., 2011; Deshmukh et al., 2013). Breuer et al. (2014) hereafter use questionnaires to measure investors’ preference for ambiguity and loss to test the influence of behavioral preference parameters on the corporate dividend policy, supporting Baker and Wurgler’s (2004) argument. Their results show that investors’ ambiguity aversion and loss aversion have a positive effect on dividends payment.

Unlike Breuer et al. (2014), based on the irrational investor perspective, we examine whether managers’ decision-making is affected by those behavioral biases. Numerous studies have examined the influence of business strategy on varied decision-making (Higgins et al., 2015; Navissi et al., 2017; Habib et al., 2018a, etc.).

Numerous studies have examined the influence of business strategy on financial report quality (Bentley et al., 2013; Habib et al., 2018b), managerial decisions (Higgins et al., 2015; Navissi et al., 2017), and the stock price crashes (Habib et al., 2018a), although there is little research examining the effect of business strategy on the dividend policy. Therefore, our research examines the impact of behavior on dividends in two aspects contemporaneously. We examine how the dividend policy changes with the attitude when managers face uncertainties and potential loss and the business strategy. Uncertainty can be divided into two well-defined, distinct parts, risk, and ambiguity, where risk is uncertain with known probabilities and ambiguity is uncertain with unknown probabilities (Nishimura and Ozaki, 2017). The typologies of business strategy are proposed by Miles et al. (1978).

The results indicate firms that face a high degree of ambiguity or risk have a lower propensity to pay dividends, but when firms face loss-averse behavior have a higher propensity to pay dividends. In the meantime, firms with aggressive strategies tend to inhibit the payout of dividends. Regarding the influences on the amount of dividend payout, the results remain unchanged except for that of loss aversion.

We perform two robustness checks for alternative dividend policy proxies. First, we explore whether the behavioral variables and business strategy trigger corporate dividend policy initiations or omissions. We find that ambiguity and risk affect the formulation of dividend policy significantly, deserving more attention. Besides, we consider the behavior of share repurchase which is another way to pay cash to shareholders (Jiang et al., 2013). We replace the dependent variable to share repurchase and find similar results with dividends per share, confirming our results are robust.

There are three additional tests for different circumstances. First, we consider the economic fluctuations caused by the financial crisis, which affect the corporate dividend policy. Thus, we divide the sample into two groups, namely before and after the financial crisis, and examine the impact of behavior foundations and business strategy on dividend policy separately. Second, we examine whether the firms’ growth opportunities distort the influence of behavior and strategy on dividends. We use Tobins’ Q as a proxy variable for growth opportunities (Adam and Goyal, 2008; Panousi and Papanikolaou, 2012) and divide the samples into High opportunities (Tobins’ *Q* > 1) and Low opportunities(Tobins’ *Q* < =1). The results show that both risk and loss have a significant impact on dividend propensity in the case of low growth opportunities. Third, we consider the effect of corporate governance. The samples are separated into two groups, high and low corporate governance. In additional tests, we find the results remain unchanged in those firms with high corporate governance or high growth opportunities.

Our results contribute to the stream of research that helps explain the association between behavioral characteristics, firm-level characteristics, executive characteristics, business strategy, and managerial decision-making (Baker and Wurgler, 2004; Breuer et al., 2014; Bhattacharya et al., 2016; Xiao et al., 2017). More importantly, we document the effects of behavioral foundation variables and strategic behavior on corporate dividend policy with irrational and rational behaviors, respectively.

The rest of this study is organized as follows. Section 2 presents a literature review and hypothesis development. Section 3 describes the data and methodology. Section4 displays the empirical results and robustness checks. Section 5 displays the additional tests. Section 6 is the conclusion.

## Literature Review and Hypothesis Development

Dividend policy is an essential component in corporate decision-making. An appropriate dividend policy enhances the development of a firm, ensures the return of shareholders’ investment, and establishes a positive prospect in the stock market (Ravid and Sudit, 1994; Harford et al., 2018). The well-known signaling hypothesis argues that firms paying cash dividends implicit future profitability (Miller and Rock, 1985; Kumar, 1988; Shapiro and Zhuang, 2015). Paying cash dividends can mitigate agency problems, such as reducing the possibility of over-investment and protecting the rights of small and medium shareholders as well as creditors (Jensen and Meckling, 1976).

However, it is difficult to formulate an optimal dividend policy in the real world. An essential implicit assumption behind the existing theories is that managers and investors are rational decision-makers pursuing expected utility maximization and ignoring the potential effect of behavioral biases on corporate dividend policy. Limited literature focuses on the relationship between behavior and dividend policy.

Corporate finance theory has recently started considering common personality traits of decision-makers and behavioral biases in managers’ decision-making (Agliardi et al., 2015). This study contemporaneously considers the behavioral foundations mentioned (ambiguity, risk, and loss) in Breuer et al. (2014) and business strategies to explore the determination of dividend payout decisions from the manager’s perspective.

### The Behavioral Foundations of Corporate Dividend Policy

#### Ambiguity Aversion

“Ambiguity” is used to refer to situations in which the decision-maker appears to be not entirely confident that his/her beliefs apply. In an ambiguous world, people are very insecure about what they know. Consequently, small slivers of information can cause price volatility, with decisions far from a standard probabilistic rule. In some cases, unrealistic pessimism characterizes the behavior of decision-makers, involving overestimating the probabilities of unfavorable events and underestimating the possibilities of favorable events. Charness and Gneezy (2010) demonstrate that people are willing to pay the price to avoid ambiguity instead of making decisions with the maximum expected utility. Trautman and Van De Kuilen (2013) perform both psychometric tests and experiments and find ambiguity aversion is the typical qualitative finding in general.

Under uncertainty, decision-makers are not sure about the likelihood of the states of nature and their valuation of option payoffs “are subject to vagueness, behavioral biases and partial ignorance” (Driouchi et al., 2015). Ilut and Schneider (2014) adopt the dispersion in analysts’ earnings forecasts to estimate the ambiguity of stock returns. Higher dispersion of analysts’ forecasts can be treated as multiple potential earnings priors, which allows us to define the ambiguity definition according to the multiple-priors utility model.

Numerous studies found managers are more likely to increase cash holding for the ambiguous future (Neamtiu et al., 2014; Breuer, 2017; etc.). Neamtiu et al. (2014) show that when managers face ambiguities in future investment payoffs, they are more likely to reduce their capital expenditures and increase their cash holdings, which may not pay the dividend payment. Ambiguity-averse managers evaluate potential projects more optimistic than that what be predicted using a rational expectations framework (Nishimura and Ozaki, 2017). As a result, they may give up opportunities for venture capital investment and hold cash for possible future uncertainties, which leads to a reduction in dividends. Agliardi et al. (2015) and Breuer et al. (2017) also find cash holdings are retained longer, and dividends are less likely to be paid when the ambiguity aversion bias is sufficiently large.

Therefore, we predict that when managers face ambiguity, they tend to decrease the dividends due to their ambiguity aversion. The hypothesis is as follow:

*Hypothesis 1:* Ambiguity aversion inhibits dividend payouts.

#### Risk Aversion

Different from ambiguity, “Risk” is used to refer to any uncertainty that can be defined based on the existing probabilistic model, which is known to the decision-maker. Several studies show risk and dividend are cause and effect (Grullon and Michaely, 2002; Hoberg and Prabhala, 2009). Hoberg and Prabhala (2009) show that firms with high perceived risk will decrease dividends. Grullon and Michaely (2002) argue that firm risk decreases after dividend increases.

A-bird-in-hand theory argues that although risky investment proceeds may yield more capital gains in the future, stockholders still expect dividend payment due to the risk-aversion behavior. Breuer et al. (2014) multinational investigation gives evidence of A-bird-in-the-hand theory; in the countries with more risk-averse investors, firms tend to pay out more dividends.

Caliskan and Doukas (2015) also find risk-averse CEOs tend to stop paying dividends. They adopt the sensitivity of the CEO’s compensation to stock return volatility for measuring the risk tolerance of the CEO. The CEO’s compensation can be interpreted as inside debt. CEOs with high inside debt imply that CEOs have lower risk tolerance and have a higher propensity to pay dividends.

Therefore, this leads to our prediction that a firm with a high degree of risk is more likely to pay dividends. The hypothesis is as follow:

*Hypothesis 2:* Risk aversion stimulates dividend payouts.

#### Loss Aversion

The prospect theory put forward by the psychologist Kahneman and Tversky (1979) is the asymmetry about profit and losses decision-making. Dividends as a deterministic gain reduce the exposure to future shocks. If there is a positive probability that future shocks cause negative returns, dividends can be utilized to reduce the exposure to potential future losses. This result is driven by the specific curvature of the value functions, which have a kink according to prospect theory, implying that avoiding losses is more important than acquiring gains of the same size.

Shiller (1998) found that people feel the pain of regret when they have made errors. Therefore, loss aversion is a case of regret aversion. Due to “loss aversion,” managers may avoid potential future losses by performing the earning management. In the meantime, managers would pay dividends to cater to investors.

Therefore, we predict, a firm with loss-averse behavior may have a strong propensity to pay dividends. The hypothesis is as follow:

*Hypothesis 3:* Loss aversion stimulates dividend payouts.

### Corporate Strategic Behavior and Dividend Policy

Although growing studies examine the influence of business strategy on financial reporting and audit fees (Bentley et al., 2013), tax aggressiveness (Higgins et al., 2015), investment efficiency (Navissi et al., 2017), stock price crash risk (Habib et al., 2018a), annual report readability (Habib et al., 2018b), there is little research examining the effect of business strategy on the dividend policy.

The well-known typologies of business strategy are proposed by Miles et al. (1978), who describe business strategy regarding prospector, defender, analyzer, and reactor. Previous studies have identified the advantages of Miles et al. (1978) and Miles and Snow’s (2003) typologies over the other typologies (Hambrick, 1983; Bentley et al., 2013). Miles et al. (1978) and Miles and Snow (2003) define prospectors as firms that follow an innovative strategy, tend to exploit new products and market opportunities. Therefore, prospectors tend to preserve liquidity against the potential costs of external financing. Magerakis and Tzelepis (2020) give evidence of prospectors may prefer to hoard cash for future investments. Unlike the prospectors, defenders are defined as firms that follow a cost leadership strategy, avoid exposure to risk and uncertainty, and maintain stability. They make decisions carefully and make products with available substitutes but do not pursue new opportunities hurriedly.

A company will weigh the costs and benefits of retained earnings when formulating specific dividend policies. On the one hand, manager tends to retain earnings instead of paying the dividend, if there are a large number of considerable growth opportunities. The manager considers the profits of the reinvestment would be higher than the cost, which is in line with the prospectors. On the other hand, because a mature company with increasing profitability has fewer investment opportunities, the cost of reinvestment would be greater than the profit. Managers tend to pay out cash dividends instead of saving earnings for new investments, which is in line with the defenders (DeAngelo et al., 2006; Huang and Paul, 2017).

Thus, using the strategy score constructed by Bentley et al. (2013), this paper investigates the effect of corporate strategic behavior on dividend policy. The hypothesis is as follow:

*Hypothesis 4:* A firm with higher strategy scores (prospectors) tends to inhibit dividend payouts.

## Data and Methodology

### Data Description

The sample comprises all publicly traded U.S. firms, which were obtained from CRSP and Compustat, covering from 2000 to 2016. To calculate the strategy score, this study backward covers sample data for 5 years. We drop financial institutions and utility firms (*SIC* 4,900–4,999 and 6,000–6,999) due to the regulated nature of these industries, delete those observations with the book-to-market ratio less than zero and winsorize all continuous variables at the 1st and 99th percentiles to remove the effect from extreme observations. Our sample includes 10,346 firm-year observations between 2000 and 2016. This paper obtained accounting data from Compustat, stock trading data from CRSP, analyst data from IBES, and the shareholder proposals and governance data from RiskMetrics.

### Measurements of Behavior Foundations

#### Ambiguity

“Ambiguity” is used to refer to situations that the decision-maker appears to be not fully confident about his/her beliefs (Agliardi et al., 2015). It is difficult to measure ambiguity. One of the methods is to use questionnaires or experiments to test the degree of perceived ambiguity. However, there are still restrictions to capture managers’ real perceptions.

Abarbanell et al. (1995) firstly argue that if forecast dispersion after an earnings announcement reflects uncertainty about firms’ future cash flows, then investors desire additional information and price fluctuates around the subsequent earnings release. Growing studies found the dispersion of financial analysts’ earnings forecasts (DISP) would be an appropriate proxy for uncertainty (Barron and Stuerke, 1998; Diether et al., 2002; Zhang, 2006; Habib et al., 2011). Neamtiu et al. (2014) indicate the dispersion of financial analysts’ earnings forecast is likely due to differences in their expectations regarding the distributions governing the forecasted data. DISP is more consistent with the definition of ambiguity than the risk in forming expectations from commonly shared information (Camerer and Weber, 1992). Neamtiu et al. (2014) found managers increase cash holding as ambiguity increases.

Therefore, this study measures the forecast dispersion as the standard deviation of analyst annual earnings forecasts in the month before the fiscal period end date divided by the absolute value of the mean forecast to represent the degree of ambiguity firms faced.

#### Risk Aversion

We use the stock’s idiosyncratic volatility as a proxy of firm risks and estimate idiosyncratic volatility relative to the Fama and French (1993) three-factor model. We estimate yearly idiosyncratic volatility values using monthly return data. Especially for year t and stock i, we estimate the following regression model:


(1) $$r_{i.t}=\beta_{0}+\beta_{MKT,i,t}+\beta_{SMB,i,t}SMB_{t}+\beta_{HML,i,t}HML_{t}+\varepsilon_{i,t}$$

In Equation (1), is the stock returns over the risk-free rate, represent the market, size, and book-to-market factors^1^, respectively. The idiosyncratic volatility of stock i during the period t is defined as the standard deviation of the residuals and estimated over the previous 12 months. $\beta_{0},\beta_{MKT,i,t},\beta_{SMB,i,t},\beta_{HML,i,t}$ represent the intercept, and the coefficients of the market, size, and book-to-market factors, respectively.

#### Loss Aversion

Several studies found that firm managers tend to achieve profitability and avoid losses through earning management. Burgstahler and Dichev (1997) provide conclusive evidence that firms systematically avoid reporting earnings losses. Degeorge et al. (1999) suggest that a corporate manager reported earnings for three purposes, namely, to avoid losses, to avoid earnings decreases, and to meet analysts’ earnings expectations. Thus, if the managers are loss aversion, they might use earning management to avoid loss. Cornett et al. (2007) use (EBIT - Discretionary Accruals)/Assets as the measure of unmanaged performance. Based on their model, we define a dummy variable “Loss” to describe the behavior of loss aversion using real earning management.

Roychowdhury (2006) and Cohen and Zarowin (2010) argue that REM is a combination of three parts, equal to abnormal production costs (ABPROD) minus abnormal discretionary expenses (ABDISX), and abnormal operating cash flow (ABCFO). To obtain REM, we estimate the following model regressions:


(2) $$\begin{array}{l} \frac{PROD_{i,t}}{AT_{i,t-1}}=\beta_{0}+\beta_{1}\frac{1}{AT_{i,j-1}}+\beta_{2}\frac{SALES_{i,t}}{AT_{i,j-1}}\\ +\beta_{3}\frac{\Delta SALES_{i,t}}{AT_{i,j-1}}+\beta_{4}\frac{\Delta SALES_{i,j-1}}{AT_{i,j-1}}+\varepsilon_{i,t} \end{array}$$

(3) $$\frac{DISX_{i,t}}{AT_{i,j-1}}=\beta_{0}+\beta_{1}\frac{1}{AT_{i,j-1}}+\beta_{2}\frac{SALES_{i,t}}{AT_{i,j-1}}+\varepsilon_{i.t}$$

(4) $$\frac{CFO_{i,t}}{AT_{i,j-1}}=\beta_{0}+\beta_{1}\frac{1}{AT_{i,j-1}}+\beta_{2}\frac{SALES_{i,t}}{AT_{i,j-1}}+\beta_{3}\frac{\Delta SALES_{i,t}}{AT_{i,j-1}}+\varepsilon_{i,t}$$

In equation (2), PROD_i,t_ the production costs of firm i in period t, AT_i,t-1_ is total assets of firm i in period t-1, $SALES_{i,t}\;$ denotes the sales of firm i in period t.SALES _i,t_ = SALES_i,t_ - SALES_i,t-1_. In equation (3), DISX_i,t_ is discretionary expenses of firm i in period t. In equation (4), CFO_i,t_ is the operating cash flow of firm i in period t. The residuals of the three regressions are ABPROD, ABDISX, and ABCFO, respectively. REM can be calculated as equation (5).


(5) REM=ABPROD−ABDISX−ABCFO

REM represents the real-earning-management for a firm. This study constructs the loss aversion proxy by “Loss.” For firm i, if its ROA > 0 and ROA-REM < 0, then the dummy variable “Loss” equals one, otherwise “Loss” equals zero.

### Measurement of Business Strategic

Following Bentley et al. (2013), we use their business strategy score to proxy for a firm’s strategic behavior. Based on Miles et al. (1978) and Miles and Snow’s (2003) framework of business strategy, Bentley et al. (2013) construct their composite STRATEGY score by using six variables which are computed using a rolling average over the prior 5 years, including (a) the ratio of research and development to sales (firm’s propensity to seek new products); (b) the ratio of employees to sales (firm’s ability to produce and distribute its goods and services efficiently); (c) a historical growth measure (one-year percentage change in total sales) (firm’s historical growth); (d) the ratio of marketing (SG&A) to sales (firm’s emphasis on marketing and sales); (e) a measure of employee fluctuations (standard deviation of total employees); and (f) a measure of capital intensity (net PPE scaled by total assets) (firm’s focus on production). Each of the six individual variables is ranked by forming a quintile within each two-digit *SIC* industry-year.

Within each firm-year observation, those observations with variables in the highest quintile are given a score of 5, in the second-highest quintile, a score of 4, and so on. The observations with variables in the lowest quintile are given a score of 1. Then by summing the six scores, we can obtain the strategy score for a firm. Higher STRATEGY scores represent companies (Prospectors) with more aggressive strategies, and lower scores represent companies (Defenders) with more conservative. In our samples, the range of the STRATEGY score is 8 to 28.

### Measurements of Dividend Payout

This study adopts two measures to represent the corporate dividend policy. First, a dummy variable “Divpayer” as cash dividend is greater than zero. Second, dividends per share represent the level of dividend payment. Therefore, we can investigate the influence of behavioral foundation and business strategy on determining dividend policy with both the propensity and amount of dividends.

Table 1 reports the definition of dependent and independent variables.

**Table 1:** Variable descriptions.

	Variable	Description
Dependent variables	DIVPAYER	The indicator variable equals one if cash dividends are greater than zero.
	DIV	Dividends per share.
Behavioral foundations	AMBIGUITY	The dispersion of financial analysts’ earnings forecasts is measured as the standard deviation of analyst annual earnings forecasts in the month before the fiscal period end date divided by the absolute value of the mean forecasts.
	LOSS	Indicator variable equal to one, if ROA > 0 and ROA-REM < 0.
	RISK	Stock’s idiosyncratic volatility, calculated by the standard deviation of the residuals of a Fama–French three factors model.
Business strategy	STRATEGY	Discrete score with values ranging from 6 to 30 where high (low) values indicate prospector (defender) firms, respectively.
Control variables	SIZE	Natural logarithm of total asset.
	LEVERAGE	Financial leverage equals total debt divided by total assets
	BM	Book-to-market ratio equal to total common equity outstanding divided by the market capitalization at the end of the fiscal year.
	INDPEND	The proportion of independent directors on the board of directors.
	INST	The percentage of outstanding shares owned by total institutional investors.
	FCF	Free cash flow, is calculated by the cash flow generated by the company’s operating activities minus capital expenditures.
	TOBIN’S Q	Market capitalization over total assets.
	GINDEX	Governance index constructed by Gompers et al. (2003) with values ranging from 0 to 24 where high (low) values indicate low (high) governance firms, respectively.
Dependent variables (for robustness check)	DIVI	Indicator variable equals one if a company pays dividends but did not in the prior year.
	DIVO	Indicator variable equals one if a company does not pay dividends but did in the prior year.
	REPURCHASE	The number of treasury shares repurchased by the company.

### Empirical Models

Following Breuer et al. (2014) and Bentley et al. (2013), we build the following models to investigate the effect of behavior foundations and business strategy on corporate dividend policy. This paper uses the Logit model in equation (6) and the OLS model in equation (7). The year- and industry-fixed effects are controlled in each model.


(6) $$\begin{array}{l} Divprayer_{i,t}=\beta_{0}+\beta_{1}Ambiguity_{i,t-1}+\beta_{2}Loss_{i,t-1}+\beta_{2}Risk_{i,t-1}\\ +\beta_{4}Strategy_{i,t-1+}\beta_{5}Size_{i,t-1}+\beta_{6}Leverage_{i,t-1}\\ +\beta_{1}BM_{i,t-1}+\beta_{8}Independ_{i,t-1}+\beta_{9}Inst_{i,t-1}\\ +\beta_{10}FCF_{i,t-1}+\beta_{11}\sum ind+\varepsilon_{i,t} \end{array}$$

(7) $$\begin{array}{l} Div_{i,t}=\beta_{0}+\beta_{1}Ambiguity_{i,t-1}+\beta_{2}Loss_{i,t-1}+\beta_{3}Risk_{i,t-1}\\ +\beta_{4}Strategy_{i,t-1}+\beta_{5}Size_{i,t-1}+\beta_{6}Leverage_{i,t-1}\\ +\beta_{7}BM_{i,t-1}+\beta_{2}Independ_{i,t-1}+\beta_{2}Inst_{i,t-1}\\ +\beta_{10}FCF_{i,t-1}+\beta_{11}\sum year+\beta_{12}\sum Ind+\varepsilon_{i,t} \end{array}$$

*Divpayer* and *Div* represent the firm that dividends payout propensity and dividend per share, respectively. The inclusion of the control variables follows the prior literature on the determinants of dividend policy. The findings of Fama and French (2001) indicate that large firms are more likely to pay dividends but contrary in the case of the firms with more investments facing great growth opportunities (also see in DeAngelo et al., 2006). Leverage is shown to be negatively associated with future dividend policy (Brockman and Unlu, 2009). Therefore, we consider company size, capital structure, and growth as control variables in the corporate dividend policy model. *Size*, *Leverage*, and BM are measured as the natural log of total assets, the total liabilities over total assets, and the book-to-market value of equity, respectively.

We then include a series of variables related to dividend policy, such as board independence and institutional ownership. *Indpend* is the proportion of independent directors on the board of directors. *Inst* is the percentage of outstanding shares owned by total institutional investors.

Within Bhattacharya et al. (2016) models, more board independence explains higher dividends, and Harford et al. (2018) also find firms pay fewer dividends when they have higher levels of institutional ownership. Besides, classical agency theory predicts that corporate managers with substantial free cash flow are more likely to invest in negative net present value projects; therefore, paying out dividends would be better for shareholders (Jensen, 1986). Therefore, we add free cash flow (*FCF*) as a control variable to exclude the effect of potential agency costs on dividend payments. All models control for industry and year-fixed effects. For the detail of variables, please refer to Table 1.

## Empirical analysis

### Descriptive Statistics

Table 2 reports the descriptive statistics. There are a slightly higher proportion of firms paying dividends (57%) in the US stock market. Regarding the level of dividend payment, the standard deviation is relatively large, which means that the number of dividends per share varies greater. The mean values of the *Ambiguity* and *Risk* are 0.073 and 25.170, respectively. *Loss* as a dummy variable, the mean value is 0.289, which means 28.9% of observations have the loss-averse behavior. The sample firms have an average strategy score of 18.714, and the max and min are 28 and 8, respectively. Besides, the firms’ size measured by the log of firms’ total assets is averagely 7.709, with a minimum of 4.743 and a maximum of 6.593. The mean book-to-market ratio is 0.477. The average proportion of institutional shareholding is 0.765. On average, the board independence ratio is 0.753. Moreover, the sample firms have a free cash flow of 0.085 on average with a high standard deviation (0.186).

**Table 2:** Descriptive statistics.

VARIABLE	MEAN	STD	MIN	Q1	MEDIAN	Q3	MAX
DIVPAYER	0.569	0.495	0.000	0.000	1.000	1.000	1.000
DIV	0.445	0.652	0.000	0.000	0.170	0.668	5.000
AMBIGUITY	0.073	0.239	0.000	0.009	0.019	0.046	3.333
LOSS	0.289	0.453	0.000	0.000	0.000	1.000	1.000
RISK	25.170	14.519	5.852	15.421	21.794	30.937	166.858
STRATEGY	18.714	2.851	8.000	17.000	19.000	21.000	28.000
SIZE	7.709	1.503	4.743	6.593	7.546	8.663	12.224
LEVERAGE	0.197	0.156	0.000	0.049	0.191	0.305	0.684
BM	0.477	0.347	0.000	0.258	0.401	0.602	7.337
INDPEND	0.753	0.138	0.000	0.667	0.778	0.875	1.000
INST	0.765	0.152	0.051	0.669	0.780	0.879	1.000
FCF	0.085	0.186	−0.710	−0.023	0.079	0.195	0.632

This table presents the mean, standard deviation (STD), minimum (Min), maximum (Max), 25 percentiles (Q1) and 75 percentiles (Q3) for all the variables used in the main tests.

Table 3 presents the Pearson (lower triangle) and Spearman (upper triangle) correlations among the main variables used in this study. The results indicate that both the Pearson and Spearman correlations are qualitatively similar, and therefore, we will discuss only the Pearson correlations for the variables. First, the relationships between behavioral foundations and dividend-paying propensity, and the amounts of dividends are similar. All the behavioral foundation variables are negatively and significantly related to the dividend policy, except for the relationship between loss and the propensity of paying dividends. Second, *Divpayer* and *Div* are significantly and positively correlated with firm size, leverage, board independence, and free cash flow, whereas negatively correlated with book-to-market ratio and institutional ownership. Third, there are weak correlations between independent variables.

**Table 3:** Correlation coefficients.

	DIVPAYER	DIV	AMBIGUITY	LOSS	RISK	STRATEGY	SIZE	LEVERAGE	BM	INDPEND	INST	FCF
DIVPAYER	1.000	0.894^***^	−0.161^***^	−0.004	−0.364^***^	−0.015	0.334^***^	0.154^***^	−0.101^***^	0.169^***^	−0.221^***^	0.055^***^
DIV	0.593^***^	1.000	−0.217^***^	−0.024^**^	−0.446^***^	−0.001	0.423^***^	0.179^***^	−0.168^***^	0.248^***^	−0.261^***^	0.092^***^
AMBIGUITY	−0.100^***^	−0.102^***^	1.000	0.013	0.381^***^	−0.027^***^	−0.192^***^	−0.042^***^	0.344^***^	−0.087^***^	0.024^**^	−0.133^***^
LOSS	−0.004	−0.035^***^	−0.009	1.000	−0.014	−0.153^***^	0.083^***^	0.023^**^	0.131^***^	0.012	0.017^*^	−0.007
RISK	−0.340^***^	−0.336^***^	0.183^***^	−0.022^**^	1.000	0.015	−0.379^***^	−0.091^***^	0.231^***^	−0.219^***^	0.065^***^	−0.096^**^
STRATEGY	−0.011	0.012	0.006	−0.163^***^	0.021^**^	1.000	0.051^***^	0.064^***^	−0.051^***^	−0.028^***^	−0.041^***^	0.087^***^
SIZE	0.345^***^	0.444^***^	−0.085^***^	0.082^***^	−0.337^***^	0.046^***^	1.000	0.381^***^	−0.101^***^	0.256^***^	−0.106^***^	0.213^***^
LEVERAGE	0.130^***^	0.138^***^	0.022^**^	0.019^**^	−0.038^***^	0.055^***^	0.332^***^	1.000	0.026^***^	0.137^***^	−0.046^***^	−0.061^***^
BM	−0.124^***^	−0.182^***^	0.179^***^	0.107^***^	0.259^***^	−0.047^***^	−0.089^***^	0.056^***^	1.000	−0.038^***^	0.070^***^	−0.386^***^
INDPEND	0.143^***^	0.215^***^	−0.013	0.012	−0.198^***^	−0.028^***^	0.223^***^	0.092^***^	−0.026^***^	1.000	0.141^***^	0.009
INST	−0.200^***^	−0.222^***^	−0.020^**^	0.016	−0.033^***^	−0.027^***^	−0.119^***^	−0.040^***^	0.005	0.202^***^	1.000	−0.007
FCF	0.047^***^	0.109^***^	−0.076^***^	−0.008	−0.085^***^	0.098^***^	0.164^***^	−0.098^***^	−0.305^***^	0.011	0.007	1.000

This table presents the Pearson and Spearman correlations for the sample observations for all the main variables used. The left triangle shows the Pearson Coefficient, and the right shows the Spearman Coefficient. ^*^, ^**^ and ^***^ mean statistical significance at 10, 5 and 1%, respectively.

### The Effect of Behavioral Foundations and Business Strategy in Corporate Dividend Policy

This study aims to examine the effect of behavioral foundations (ambiguity, loss, and risk) and corporate strategic behavior on dividend policy. Panel A of Table 4 presents the empirical results based on the dependent variable is dividends payout propensity.

**Table 4:** The influence of behavior foundations and business strategy on dividend policy.

	(1)	(2)	(3)	(4)	(5)	(6)
**Panel A Y=Divpayer**						
Intercept	−0.100	−0.037	−0.073	2.363	1.960	4.117
	(0.00)	(0.00)	(0.00)	(0.08)	(0.06)	(0.13)
Ambiguity		−0.577^***^				−0.365^***^
		(−4.36)				(−2.81)
Loss			0.273^***^			0.175^**^
			(4.14)			(2.54)
Risk				−0.051^***^		−0.049^***^
				(−17.02)		(−16.21)
Strategy					−0.112^***^	−0.097^***^
					(−10.56)	(−8.91)
Size	0.661^***^	0.654^***^	0.654^***^	0.521^***^	0.684^***^	0.536^***^
	(24.81)	(24.50)	(24.47)	(18.51)	(25.15)	(18.64)
Leverage	−1.908^***^	−1.848^***^	−1.938^***^	−1.466^***^	−1.829^***^	−1.395^***^
	(−8.15)	(−7.88)	(−8.27)	(−6.10)	(−7.73)	(−5.74)
bm	−1.051^***^	−0.993^***^	−1.117^***^	−0.762^***^	−1.069^***^	−0.788^***^
	(−10.06)	(−9.43)	(−10.50)	(−7.10)	(−10.16)	(−7.17)
Indpend	1.799^***^	1.819^***^	1.796^***^	1.845^***^	1.643^***^	1.724^***^
	(7.40)	(7.46)	(7.37)	(7.36)	(6.72)	(6.85)
Inst	−2.767^***^	−2.796^***^	−2.757^***^	−3.270^***^	−2.779^***^	−3.292^***^
	(−11.82)	(−11.92)	(−11.77)	(−13.33)	(−11.73)	(−13.24)
FCF	−0.222	−0.245	−0.194	−0.176	−0.049	−0.016
	(−0.91)	(−1.00)	(−0.79)	(−0.70)	(−0.20)	(−0.06)
Year	YES	YES	YES	YES	YES	YES
Ind	YES	YES	YES	YES	YES	YES
Pseudo *R*^2^	43.39%	43.51%	43.49%	45.25%	44.02%	45.80%
*N*	10,346	10,346	10,346	10,346	10,346	10,346
**Panel B Y=Div**						
Intercept	−0.186^*^	−0.178	−0.192^*^	0.027	0.010	0.193
	(−1.67)	(−1.59)	(−1.72)	(0.23)	(0.08)	(1.58)
Ambiguity		−0.066^***^				−0.047^***^
		(−3.75)				(−2.72)
Loss			−0.013			−0.026^**^
			(−1.14)			(−2.21)
Risk				−0.005^***^		−0.004^***^
				(−12.38)		(−11.84)
Strategy					−0.012^***^	−0.011^***^
					(−6.16)	(−5.79)
Size	0.151^***^	0.150^***^	0.152^***^	0.136^***^	0.153^***^	0.138^***^
	(27.96)	(27.73)	(27.91)	(24.30)	(28.36)	(24.72)
Leverage	−0.228^***^	−0.220^***^	−0.227^***^	−0.184^***^	−0.216^***^	−0.166^***^
	(−5.44)	(−5.24)	(−5.40)	(−4.40)	(−5.13)	(−3.95)
bm	−0.227^***^	−0.220^***^	−0.224^***^	−0.199^***^	−0.227^***^	−0.191^***^
	(−13.47)	(−13.19)	(−13.23)	(−12.31)	(−13.50)	(−11.81)
indpend	0.308^***^	0.311^***^	0.308^***^	0.305^***^	0.300^***^	0.298^***^
	(8.45)	(8.51)	(8.44)	(8.39)	(8.21)	(8.20)
Inst	−0.616^***^	−0.621^***^	−0.615^***^	−0.651^***^	−0.614^***^	−0.650^***^
	(−14.24)	(−14.34)	(−14.23)	(−15.02)	(−14.26)	(−15.07)
FCF	0.092^*^	0.089^*^	0.090^*^	0.083^*^	0.114^**^	0.097^**^
	(1.94)	(1.87)	(1.90)	(1.74)	(2.37)	(2.04)
Year	YES	YES	YES	YES	YES	YES
IND	YES	YES	YES	YES	YES	YES
*R* ^2^	48.15%	48.20%	48.16%	48.74%	48.37%	48.97%
Adj *R*^2^	46.53%	46.58%	46.53%	47.14%	46.75%	47.36%
*N*	10,346	10,346	10,346	10,346	10,346	10,346

Panel A presents the results of the logistic regression in which the dependent variable equals one if the firm pays dividends at time *t*, and zero otherwise. Panel B presents the results of the OLS regression in which the dependent variable is dividends per share at time *t*. t-value is presented in the parentheses. Model (1) includes only control variables, Model (2) to (5) examine the effects of 3 behavioral variables and strategy scores separately, and Model (6) incorporates all variables. All models include industry and year dummies. ^*^, ^**^ and ^***^ represent the statistical significance at 10, 5 and 1%, respectively.

In model (1), we include all control variables to test whether those factors influence the corporate decision of paying the dividend. In model (2)–(5), we add behavioral variables-Ambiguity, Loss, Risk separately. Finally, model (6) estimates the propensity to pay dividends contemporaneously, after considering all the behavioral variables.

The first model shows that firms with a larger size and higher board independence are likely to pay dividends, where leverage, book-to-market ratio, institution ownerships, and free cash flow decrease the propensity to pay dividends. Models (2)–(5) show all behavioral variables significantly affect the propensity to pay dividends at the 1% significance level. The degree of ambiguity is significantly and negatively related to the propensity of dividends.

Previous studies found managers are more likely to increase cash holding for the ambiguous future (Neamtiu et al., 2014; Agliardi et al., 2015; Breuer, 2017; Nishimura and Ozaki, 2017, etc.) Similarly, our results show the negative influence of ambiguity on cash holding policy. When the degree of ambiguity is high, the firm manager prefers to save cash instead of paying dividends for the uncertain future. The results are consistent with hypothesis one.

The results of column (3) of Table 4 present a positive relationship between loss-aversion and dividend payout policy, which means that the loss-aversion managers would tend to pay dividends. Our results are consistent with the argument of Shiller (1998). Shiller (1998) found loss aversion is a case of regret aversion. Managers may avoid potential future losses by paying dividends to cater to investors.

However, model (4) of Table 4 shows that a company with a high degree of risk omits to pay dividends, which is violent with our hypothesis 2. Our results are consistent with the findings of Hoberg and Prabhala (2009) and Caliskan and Doukas (2015), managers decrease or stop dividend payout when they face corporate risks. Companies with high risks are often accompanied by better growth opportunities and future returns. Therefore, managers prefer to reinvest with retained earnings for pursuing higher growth and performance in the future.

Business strategic behavior is found to be negatively related to the dividend payout. The higher the strategy score, the less propensity to pay dividends. A higher business strategy score means the firm would carry out a prospecting strategy, which leads to more financing needs to retain profits instead of paying dividends. We find there is supporting evidence for our hypothesis in models (6), incorporating both behavioral dimensions and business strategy.

Panel B of Table 4 reports the relationships between behavioral variables and the amount of dividend payment after controlling for the potential determinants of dividend policy. The results regarding the amount of dividend payment are similar to dividend payout propensity. The coefficients of ambiguity, risk, and business strategy are significantly negative, suggesting that a firm with a high degree of ambiguity, risk, or a high strategy score would decrease dividends. However, the influence of loss aversion on dividend payments is different from the case of dividend propensity. As we discussed above, a loss-averse firm tends to pay dividends but decreases the number of dividends.

### Robustness Check: The Influence of Behavioral Foundations and Business Strategy on Dividend Initiations and Omissions

To verify the robustness of our results, we further consider a possible endogeneity problem. In this subsection, we examine the influence of behavioral foundation and business strategy on the initiate/omit dividend payout. Initiating or omitting to pay dividends is an important decision for a company. The change of dividend policy will have a significant impact on a firm’s stock price. Firm managers often make decisions carefully, especially when the decisions are highly related to stock prices. Therefore, whether a firm initiates paying dividends deserves us to pay more attention to it.

In Panel A of Table 5, we find strong evidence that ambiguity aversion, risk aversion, and strategy scores negatively affect the dividends initiation, except for loss-averse behavior. Columns (1), (2), (3), and (4) show that the coefficients of behavior proxies and strategy scores are all negative and significant except for Loss, where column (5) shows a similar result. The results are consistent with that of the dividends propensity.

**Table 5:** The influence of behavioral foundations and business strategy on the dividend initiations and omissions.

	(1)	(2)	(3)	(4)	(5)
**Panel A: Dividend Initiations**					
Intercept	−4.990	−5.160	−4.232	−4.182	−3.300
	(−0.01)	(−0.01)	(−0.01)	(−0.01)	(−0.01)
Ambiguity	−1.185^*^				−1.031^*^
	(−1.95)				(−1.78)
Loss		−0.058			−0.106
		(−0.31)			(−0.57)
Risk			−0.017^***^		−0.015^**^
			(−2.60)		(−2.34)
Strategy				−0.051^*^	−0.049^*^
				(−1.82)	(−1.73)
Size	0.286^***^	0.297^***^	0.250^***^	0.297^***^	0.254^***^
	(3.73)	(3.87)	(3.20)	(3.89)	(3.23)
Leverage	−2.455^***^	−2.594^***^	−2.413^***^	−2.563^***^	−2.290^***^
	(−3.54)	(−3.75)	(−3.46)	(−3.70)	(−3.26)
bm	−0.044	−0.131	−0.030	−0.160	0.060
	(−0.16)	(−0.46)	(−0.10)	(−0.57)	(0.21)
Indpend	0.110	0.094	0.111	0.057	0.094
	(0.16)	(0.14)	(0.16)	(0.08)	(0.14)
Inst	−1.025	−0.910	−1.188^*^	−0.910	−1.241^*^
	(−1.54)	(−1.38)	(−1.77)	(−1.38)	(−1.84)
FCF	0.377	0.394	0.413	0.493	0.444
	(0.53)	(0.56)	(0.57)	(0.69)	(0.62)
Ind	YES	YES	YES	YES	YES
Year	YES	YES	YES	YES	YES
Pseudo *R*^2^	7.62%	7.50%	7.65%	7.57%	7.81%
*N*	4,614	4,614	4,614	4,614	4,614
**Panel B. Dividend omissions**					
Intercept	−1.540	−1.761	−3.632	−2.123	−3.761
	(−0.10)	(−0.12)	(−0.27)	(−0.15)	(−0.24)
Ambiguity	0.912^***^				0.815^**^
	(2.75)				(2.47)
Loss		−0.301			−0.334
		(−0.86)			(−0.91)
Risk			0.043^***^		0.041^***^
			(3.72)		(3.53)
Strategy				0.035	0.011
				(0.60)	(0.19)
Size	−1.260^***^	−1.289^***^	−1.178^***^	−1.320^***^	−1.134^***^
	(−6.04)	(−6.18)	(−5.46)	(−6.27)	(−5.25)
Leverage	4.637^***^	4.807^***^	4.427^***^	4.707^***^	4.333^***^
	(3.74)	(3.92)	(3.57)	(3.82)	(3.46)
bm	1.544^***^	1.779^***^	1.566^***^	1.706^***^	1.456^***^
	(4.03)	(4.61)	(4.15)	(4.49)	(3.74)
Indpend	−3.496^***^	−3.189^**^	−2.936^**^	−3.194^**^	−3.128^**^
	(−2.78)	(−2.55)	(−2.31)	(−2.56)	(−2.43)
Inst	−0.124	0.237	0.221	0.235	−0.135
	(−0.10)	(0.20)	(0.19)	(0.20)	(−0.11)
FCF	−2.554^**^	−2.688^**^	−2.620^**^	−2.816^**^	−2.530^*^
	(−2.03)	(−2.20)	(−2.07)	(−2.27)	(−1.91)
Ind	YES	YES	YES	YES	YES
Year	YES	YES	YES	YES	YES
Pseudo *R*^2^	7.41%	7.29%	7.50%	7.29%	7.62%
*N*	5,761	5,761	5,761	5,761	5,761

Panel A presents the results of the logistic regression in which the dependent variable equals one if the firm pays dividends at time *t* but did not in time *t*-1, and zero otherwise. Panel B reports the results of the logistic regression in which the dependent variable equals one if the firm does not pay dividends at time t but did in time t-1, and zero otherwise. t-value is presented in parentheses. Model (1) to (4) examine the effects of three behavioral variables and strategy scores separately. Model (5) incorporates all variables. All models include industry and year dummies. ^*^, ^**^ and ^***^ represent the statistical significance at 10, 5 and 1%, respectively. ^*^, ^**^ and ^***^ mean statistical significance at 10, 5 and 1%, respectively.

In Panel B of Table 5, we also find strong evidence that ambiguity and risk have positive effects on the dividends omission but see little evidence showing that firms with higher strategy scores or a loss-averse behavior are likely to omit dividends. Our empirical results show ththatoss-averse behavior and corporate strategic behavior are insufficient to affect the changes in dividend policy.

The change of dividend policy is an essential decision for the company, dividend initiations have positive announcement effects, but associated dividend omissions may have a negative effect (Allen and Michaely, 2003). The change of dividend policy will have a significant impact on a firm’s stock price. This study finds that the degree of ambiguity and risk affect the formulation of managerial decisions significantly, deserving more attention.

### Robustness Check: The Influence of Behavioral Foundations and Business Strategy on Share Repurchases

Stock repurchase policy, in some cases, is the substitution of dividend payout (Grullon and Michaely, 2002). Although US corporations have overwhelmingly preferred to pay dividends in the form of cash for decades, share repurchase activity has experienced extraordinary growth nowadays. Therefore, taking the ratio of the amount of repurchased treasury shares to the total assets as the dependent variable, we examine the effects of behavioral variables and strategy scores on share repurchases.

As a substitution of cash dividends (Jiang et al., 2013), the behavioral variables and strategy score show a negative effect on share repurchases. A firm suffering a high degree of ambiguity and risk would not perform repurchase shares. It should be noted that loss aversion negatively relates to the share repurchases. The results of Table 6 are mainly consistent with that of dividends per share except for the effects of loss aversion.

**Table 6:** The effect of behavior foundations and business strategy on share repurchases.

	(1)	(2)	(3)	(4)	(5)
Intercept	−1.322	−1.685^**^	−0.066	0.394	1.467
	(−1.58)	(−2.01)	(−0.08)	(0.44)	(1.61)
Ambiguity	−0.747^***^				−0.628^***^
	(−3.68)				(−3.15)
Loss		−0.557^***^			−0.673^***^
		(−4.47)			(−5.34)
Risk			−0.029^***^		−0.027^***^
			(−5.97)		(−5.56)
Strategy				−0.110^***^	−0.115^***^
				(−5.35)	(−5.53)
Size	0.359^***^	0.391^***^	0.275^***^	0.386^***^	0.311^***^
	(6.88)	(7.47)	(5.00)	(7.43)	(5.66)
Leverage	−6.260^***^	−6.285^***^	−6.071^***^	−6.237^***^	−5.822^***^
	(−13.54)	(−13.63)	(−13.08)	(−13.55)	(−12.56)
bm	−1.749^***^	−1.720^***^	−1.644^***^	−1.825^***^	−1.476^***^
	(−9.04)	(−8.90)	(−8.42)	(−9.36)	(−7.79)
Indpend	2.113^***^	2.073^***^	2.063^***^	2.008^***^	1.986^***^
	(4.13)	(4.06)	(4.05)	(3.94)	(3.90)
Inst	1.206^***^	1.276^***^	1.040^**^	1.278^***^	1.045^**^
	(2.65)	(2.81)	(2.28)	(2.81)	(2.30)
FCF	8.597^***^	8.552^***^	8.571^***^	8.831^***^	8.658^***^
	(15.39)	(15.34)	(15.32)	(15.77)	(15.51)
Ind	YES	YES	YES	YES	YES
Year	YES	YES	YES	YES	YES
*R* ^2^	26.45%	26.51%	26.64%	26.58%	27.07%
Adj *R*^2^	24.15%	24.21%	24.35%	24.29%	24.76%
*N*	10,346	10,346	10,346	10,346	10,346

This table presents the results of the OLS regression in which the dependent variable is share repurchases. t-value is presented in the parentheses. Models (1)–(4) examine the effect of each behavioral variable separately. Model (5) incorporates all variables. All models include industry and year dummies. ^*^, ^**^ and ^***^ represent the statistical significance at 10, 5 and 1%, respectively.

## Additional Tests

### The Effects of the Financial Crisis

There is a tremendous impact on the US financial market and economy during the financial crisis in 2008. During the financial crisis, a firms’ profitability drops sharply, and the future cash flows experience severe uncertainties (Campello et al., 2010). Bildik et al. (2015) find that the dividend payouts of the US-listed companies decrease significantly during the financial crisis. Therefore, this study further tests the effects of the financial crisis. The sample is divided into two groups, namely before and after the financial crisis. We perform the regression with different sample groups.

Table 7 shows the results regarding dividend payments remain unchanged even considering the effect of the financial crisis. However, the effects of ambiguity and loss-averse on the propensity to pay cash dividends are no longer significant after the financial crisis. Our results represent the effects of risk aversion and business strategy on dividend payout policy are relatively higher after the crisis.

**Table 7:** The effect of behavior foundations and business strategy on the dividend policy before and after the financial crisis.

	Y=DIVPAYER		Y=DIV	
	Before	After	Before	After
Intercept	2.959	5.402	−0.046	0.224
	(0.07)	(0.14)	(−0.58)	(1.03)
Ambiguity	−0.418^**^	−0.147	−0.046^***^	−0.066^**^
	(−2.03)	(−0.81)	(−2.74)	(−2.09)
Loss	0.184^*^	0.120	−0.012	−0.032
	(1.67)	(1.26)	(−1.09)	(−1.63)
Risk	−0.046^***^	−0.050^***^	−0.003^***^	−0.006^***^
	(−9.80)	(−11.43)	(−7.89)	(−9.18)
Strategy	−0.097^***^	−0.106^***^	−0.004^**^	−0.016^***^
	(−5.32)	(−7.21)	(−2.30)	(−5.24)
Size	0.661^***^	0.440^***^	0.108^***^	0.161^***^
	(13.57)	(11.35)	(19.38)	(17.65)
Leverage	−1.899^***^	−0.986^***^	−0.160^***^	−0.220^***^
	(−4.75)	(−2.93)	(−4.02)	(−3.06)
bm	−0.752^***^	−0.855^***^	−0.092^***^	−0.223^***^
	(−4.08)	(−5.60)	(−5.65)	(−7.74)
Indpend	1.560^***^	2.610^***^	0.289^***^	0.314^***^
	(4.66)	(5.80)	(9.51)	(3.65)
Inst	−2.948^***^	−3.801^***^	−0.384^***^	−0.891^***^
	(−8.01)	(−10.05)	(−10.24)	(−11.03)
FCF	0.103	0.088	−0.160^***^	0.277^***^
	(0.23)	(0.26)	(−3.57)	(3.59)
Year	YES	YES	YES	YES
Ind	YES	YES	YES	YES
Pseudo *R*^2^/Adj *R*^2^	50.46%	45.73%	57.20%	47.62%
*N*	4,700	5,646	4,700	5,646

This table presents the results of an additional test for the effect of the financial crisis. The sample is divided into two groups, namely after (the firm-year after 2008) and before (observations with the firm-year before 2008). The first two columns using logistic regressions, where the dependent variable equals one if the firm pays dividends and zero otherwise. The last two columns using OLS regressions, where the dependent variable is the dividends per share. We estimate all regression models using industry (two-digit *SIC* codes) and year dummy variables. The t-value is reported in parentheses. ^*^, ^**^ and ^***^ mean statistical significance at 10, 5 and 1%, respectively.

### Growth Opportunities

A company’s growth opportunities might affect the formulation of dividend policies. Managers may retain earnings to invest in potentially high-growth opportunities projects instead of paying dividends (DeAngelo et al., 2006). Therefore, this study further investigates whether the growth opportunities distort the results. Adam and Goyal (2008) evaluate the proxy variables for a firm’s growth opportunity set, and the results show that Tobin’s Q outperforms other variables. Thus, we adopt Tobin’s Q as the proxy variable for company growth opportunities. We divide the samples into two groups: high-growth companies (Tobin’s *Q* > 1) and low-growth companies (Tobin’s *Q* ≤ 1). Table 8 shows the effects of all behavioral variables and strategy scores on the dividends paying propensity for those high growth opportunity firms are similar to our main results. However, the impacts of ambiguity aversion and strategy score are not significant in the group of low growth opportunities.

**Table 8:** The effect of behavior foundations and business strategy on the dividend policy with high/low growth opportunities.

	Y=DIVPAYER		Y=DIV	
	High	Low	High	Low
Intercept	4.928	−9.708	0.253^**^	0.016
	(0.16)	(−0.13)	(2.03)	(0.08)
Ambiguity	−0.477^***^	0.067	−0.062^***^	−0.027
	(−3.17)	(0.19)	(−3.04)	(−1.42)
Loss	0.144^**^	0.893^**^	−0.016	−0.032
	(2.01)	(2.21)	(−1.33)	(−0.81)
Risk	−0.051^***^	−0.039^***^	−0.005^***^	−0.001
	(−16.00)	(−3.09)	(−11.20)	(−1.51)
Strategy	−0.107^***^	−0.039	−0.013^***^	−0.008
	(−9.37)	(−0.61)	(−6.20)	(−1.50)
Size	0.525^***^	1.028^***^	0.142^***^	0.035^**^
	(17.67)	(4.43)	(24.10)	(2.34)
Leverage	−1.346^***^	−2.916^*^	−0.190^***^	−0.269^***^
	(−5.34)	(−1.74)	(−4.34)	(−2.63)
bm	−0.846^***^	−0.720^*^	−0.318^***^	−0.053^***^
	(−4.93)	(−1.82)	(−10.91)	(−3.26)
indpend	1.621^***^	4.497^**^	0.283^***^	0.181
	(6.20)	(2.44)	(7.41)	(1.41)
Inst	−3.526^***^	−0.525	−0.650^***^	−0.093
	(−13.47)	(−0.36)	(−14.28)	(−0.96)
FCF	0.097	−2.169	0.058	0.069
	(0.36)	(−1.45)	(1.13)	(0.53)
Year	YES	YES	YES	YES
Ind	YES	YES	YES	YES
Pseudo *R*^2^/Adj *R*^2^	45.71%	56.75%	47.72%	44.52%
*N*	9,728	618	9,728	618

This table presents the results of an additional test for the effect of growth opportunities. The sample is divided into two groups, namely High (Tobin’s *Q* > 1) and Low (Tobin’s *Q* ≤ 1). The first two columns using logistic regressions, where the dependent variable equals one if the firm pays dividends and zero otherwise. The last two columns using OLS regressions, where the dependent variable is the dividends per share. We estimate all regression models using industry (two-digit *SIC* codes) and year dummy variables. The t-value is reported in parentheses. ^*^, ^**^ and ^***^ mean statistical significance at 10, 5 and 1%, respectively.

### Corporate Governance

Jensen and Meckling (1976) propose that paying dividends can inhibit the overinvestment of managers, which helps to mitigate the agency problem. Our findings also suggest that activist shareholders prefer to collect dividends in the years following the campaigns, arguably as a risk management mechanism. This study adopts the “governance index (gindex)” constructed by Gompers et al. (2003) with values ranging from 0 to 24. A high (low) governance index indicates low (high) governance firms. The sample was divided into two groups according to the degree of corporate governance. High is an indicator representing gindex less than 12 and Low representing gindex more than 13.

Table 9 shows the effects of behavioral variables and strategy scores on dividend policy with the levels of corporate governance. Although there is little evidence of the main results in low governance firms, the main results of high governance firms still hold. Therefore, the influence of behavior foundation and business strategy on dividend payout polity is significant in those high governance firms.

**Table 9:** The effect of behavior foundations and business strategy on the dividend policy with high/low corporate governance.

	Y=DIVPAYER		Y=DIV	
	High	Low	High	Low
Intercept	4.330	0.651	0.269^**^	−0.816^**^
	(0.15)	(0.01)	(2.15)	(−2.43)
Ambiguity	−0.394^***^	1.012	−0.044^**^	−0.072
	(−2.87)	(1.36)	(−2.45)	(−1.04)
Loss	0.165^**^	0.657	−0.028^**^	0.031
	(2.35)	(0.89)	(−2.30)	(0.77)
Risk	−0.049^***^	−0.038	−0.004^***^	−0.003^***^
	(−15.87)	(−1.22)	(−11.11)	(−3.23)
Strategy	−0.096^***^	−0.442^***^	−0.012^***^	<0.001
	(−8.59)	(−2.98)	(−5.92)	(−0.07)
Size	0.520^***^	1.713^***^	0.136^***^	0.192^***^
	(17.79)	(2.69)	(23.34)	(7.48)
Leverage	−1.336^***^	−6.571^**^	−0.173^***^	−0.081
	(−5.37)	(−2.28)	(−3.96)	(−0.48)
bm	−0.782^***^	−2.912^*^	−0.206^***^	−0.152^***^
	(−6.88)	(−1.67)	(−11.97)	(−2.81)
Indpend	1.610^***^	4.533^*^	0.264^***^	0.519^***^
	(6.17)	(1.66)	(6.95)	(3.60)
Inst	−3.192^***^	0.082	−0.631^***^	−0.628^***^
	(−12.50)	(0.03)	(−14.02)	(−3.27)
FCF	−0.184	−1.542	0.095^*^	−0.234
	(−0.70)	(−0.51)	(1.91)	(−0.91)
Year	YES	YES	YES	YES
Ind	YES	YES	YES	YES
Pseudo *R*^2^/Adj *R*^2^	45.65%	58.24%	47.50%	70.99%
*N*	9,692	654	9,692	654

This table presents the results of an additional test checking for the effect of corporate governance. The samples are divided into two groups, namely High (Gindex ≤12) and Low (Gindex >13). The first two columns using logistic regressions, where the dependent variable equals one if the firm pays dividends and zero otherwise. The last two columns using OLS regressions, where the dependent variable is the dividends per share. We estimate all regression models using industry (two-digit *SIC* codes) and year dummy variables. The t-value is reported in parentheses. ^*^, ^**^ and ^***^ mean statistical significance at 10, 5 and 1%, respectively.

## Conclusion and discussion

Most of the prior research focuses on explaining dividend policy from the company characteristics but is rare considering the managers’ irrational behaviors. This paper investigates the relationship between behavioral variables and business strategy and dividend policies for providing a new viewpoint on dividend policy. We consider the behavior variables in two aspects. First, we follow Breuer et al.’s (2014) method to measure the degree of ambiguity, risk, and the behavior of loss aversion and discuss whether those behavioral biases affect managers formulating the corporate dividend. Our empirical results show that firms with a high degree of ambiguity and risk are unlikely to pay cash dividends. However, those firms with loss-averse behavior tend to pay dividends. Second, we further explore whether corporate strategic behavior influences dividend policy. Adopting the strategy score constructed by Bentley et al. (2013), we find prospect companies tend not to pay cash dividends.

In the existing studies, behavioral variables are often measured by questionnaires or experiments. Most of the participants in the questionnaires and experiments are college students. The data collected from those questionnaires or experiments may not comprehensively represent the real situation. We adopt financial data to describe the managers’ behavior. Our research provides another explanation for the corporate dividend policy from the perspective of behavior.

Moreover, our study focuses on dividend policy that financial managers consider as important as the investment policy (Brav et al., 2005). Dividend payments involve large sums of money interconnected with essential financial decisions regarding real investments, issues of debt and equity, mergers and acquisitions, and the retention of earnings (Allen and Michaely, 2003). Our empirical results enhance the understanding what the effect of behavioral variables and business strategy on dividend policy is.

Although our study focuses on US firms, it is valuable for further research to compare the situation in European, Asian conditions. The characteristics of each country play a role in the management philosophy and operating style of a firm (Lu et al., 2020; Dang et al., 2021). Furthermore, the influence of stakeholders’ power on dividend policy is also an important issue. The latest study, Barros et al. (2021) found activist shareholders play a role in the policy of dividend payout. Therefore, the determinants of dividend policy are still lacking a conclusion.

## Data Availability Statement

The original contributions presented in the study are included in the article/supplementary material, further inquiries can be directed to the corresponding author.

## Author Contributions

W-JL and Y-EL developed the research framework, X-ZL performed the data processing and model estimating. W-JL, Y-EL, and H-HC contributed to the final version of the manuscript.

## Funding

This work was supported by Fundamental Research Funds of Newhuadu Business School, Minjiang University.

## Conflict of Interest

The authors declare that the research was conducted in the absence of any commercial or financial relationships that could be construed as a potential conflict of interest.

## Publisher’s Note

All claims expressed in this article are solely those of the authors and do not necessarily represent those of their affiliated organizations, or those of the publisher, the editors and the reviewers. Any product that may be evaluated in this article, or claim that may be made by its manufacturer, is not guaranteed or endorsed by the publisher.

## Footnotes

^1^We obtain factor return from Ken French’s Web Site.http://mba.tuck.dartmouth.edu/pages/faculty/ken.french/data_library.html.
